# Quantitative Measurement of Pneumothorax Using Artificial Intelligence Management Model and Clinical Application

**DOI:** 10.3390/diagnostics12081823

**Published:** 2022-07-29

**Authors:** Dohun Kim, Jae-Hyeok Lee, Si-Wook Kim, Jong-Myeon Hong, Sung-Jin Kim, Minji Song, Jong-Mun Choi, Sun-Yeop Lee, Hongjun Yoon, Jin-Young Yoo

**Affiliations:** 1 Department of Thoracic and Cardiovascular Surgery, College of Medicine, Chungbuk National University Hospital, Chungbuk National University, Cheongju 28644, Korea; mwille@chungbuk.ac.kr (D.K.); ksw713@chungbuk.ac.kr (S.-W.K.); hongjm@chungbuk.ac.kr (J.-M.H.); 2 Deepnoid, Inc., Seoul 08376, Korea; fortress544@deepnoid.com (J.-H.L.); ilkh0117@deepnoid.com (J.-M.C.); sunlee@deepnoid.com (S.-Y.L.); hyoon@deepnoid.com (H.Y.); 3 Department of Radiology, College of Medicine, Chungbuk National University Hospital, Chungbuk National University, Cheongju 28644, Korea; sjkim@chungbuk.ac.kr (S.-J.K.); minji5c579@gmail.com (M.S.)

**Keywords:** pneumothorax, artificial intelligence, deep learning, true label

## Abstract

Artificial intelligence (AI) techniques can be a solution for delayed or misdiagnosed pneumothorax. This study developed, a deep-learning-based AI model to estimate the pneumothorax amount on a chest radiograph and applied it to a treatment algorithm developed by experienced thoracic surgeons. U-net performed semantic segmentation and classification of pneumothorax and non-pneumothorax areas. The pneumothorax amount was measured using chest computed tomography (volume ratio, gold standard) and chest radiographs (area ratio, true label) and calculated using the AI model (area ratio, predicted label). Each value was compared and analyzed based on clinical outcomes. The study included 96 patients, of which 67 comprised the training set and the others the test set. The AI model showed an accuracy of 97.8%, sensitivity of 69.2%, a negative predictive value of 99.1%, and a dice similarity coefficient of 61.8%. In the test set, the average amount of pneumothorax was 15%, 16%, and 13% in the gold standard, predicted, and true labels, respectively. The predicted label was not significantly different from the gold standard (*p* = 0.11) but inferior to the true label (difference in MAE: 3.03%). The amount of pneumothorax in thoracostomy patients was 21.6% in predicted cases and 18.5% in true cases.

## 1. Introduction

Pneumothorax is a condition that requires intervention with needle aspiration or chest intubation. Its recurrence can compromise health-related quality of life (HRQoL) due to repeated urgent visits to the emergency room or hospital [1,2]. Timely diagnosis and management are vital to reduce unnecessary mortality or morbidity of pneumothorax, especially among patients affected by tension pneumothorax [3,4]. Unlike primary pneumothorax in young adults, secondary pneumothorax caused by pulmonary disease can be critical, because affected patients usually have a poor pulmonary function and fragile lung parenchyma of emphysema [2,5]. Moreover, early diagnosis and timely management of pneumothorax are essential for improving survival and HRQoL if patients are under mechanical ventilation or in a pneumonectomized state [6,7,8,9]. However, human errors, including delayed identification or misdiagnosis of pneumothorax, can develop into tension pneumothorax, with fatal consequences [10,11,12].

Artificial intelligence (AI) can help solve these problems. Well-trained AI, with high-quality data and algorithms, has been adopted in medicine [13,14]. If AI is applied for the early diagnosis of pneumothorax and treatment recommendations, delayed diagnosis can be resolved. However, apart from detecting the presence of pneumothorax, it is essential to accurately determine the amount in order to decide on a treatment method [15,16]. Chest intubation with a large bore can be the most effective treatment; however, it can cause chest pain, bleeding, shock, and other critical complications [16,17]. Moreover, when the amount of pneumothorax is less, observation or supply of oxygen is sufficient [15,18]. Therefore, AI, which can determine the presence and amount of pneumothorax, can suggest a treatment policy based on an algorithm developed by experienced thoracic surgeons. It prevents unnecessary human errors and improves the HRQoL of patients.

In this study, an AI-based management model was designed by analyzing chest radiograph and computed tomography (CT) images of patients with pneumothorax and comparing it with a clinical algorithm developed by experienced thoracic surgeons.

## 2. Methods

### 2.1. Patient Selection

This study was approved by the Institutional Review Board of Chungbuk National University Hospital (CBNUH 2020-04-030). The need for informed consent was waived by our ethics committee since data were anonymized and aggregated before access and analysis. We retrospectively evaluated 317 patients diagnosed with pneumothorax by a qualified thoracic surgeon or experienced radiologist at a tertiary referral university hospital between December 2015 and September 2020. We included those who underwent serial chest radiographs and CT and those investigated for thoracostomy decision-making and insertion processes. However, we excluded cases where an identifiable cause of pneumothorax, such as interstitial lung disease (*n* = 22) or trauma (*n* = 2), existed, where simple chest radiography or chest CT was not performed (*n* = 170), or where interpretation was difficult because of poor image quality (*n* = 9). Additionally, 14 cases managed by physicians other than thoracic surgeons were excluded. Furthermore, four patients with images that could not be processed in the AI model were excluded.

### 2.2. Hypothesis and Operational Definition

**Figure 1:**
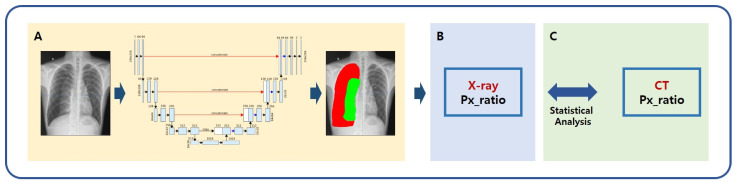
Schematic of AI modeling study for the measurement of pneumothorax. (**A**) A deep learning framework using the U-Net architecture segmented lung and pneumothorax on chest radiographs; (**B**) Quantitative value of pneumothorax was calculated using the predicted label; (**C**) The pneumothorax values calculated from the predicted label and gold standard were compared. The pneumothorax value (volume ratio) was defined as the gold standard based on a CT image. The pneumothorax value (area ratio) based on a simple chest image was defined as a true label. The two values were derived from manually segmented labels, while AI was used to derive the predicted label of pneumothorax value (area ratio) on simple chest images. X-ray = simple chest radiography, Px_ratio = pneumothorax ratio, CT = chest computed tomography.

We hypothesized that the amount of pneumothorax derived by AI modeling is not statistically different from the actual pneumothorax value. The amount of pneumothorax is calculated as the radiolucent area between the lung parenchyma and the chest wall on simple chest images and as the radiolucent volume within the pleural cavity on chest CT. The volume is more accurate; however, the area is more practical because chest CT is more expensive and time-consuming than simple radiography. In this study, pneumothorax value (volume ratio) based on a CT image was defined as the gold standard, and pneumothorax value (area ratio) based on a simple chest image was defined as the true label. The two values were derived from manually segmented labels, while AI was used to derive the predicted label of the pneumothorax value (area ratio) on simple chest images (Figure 1).

### 2.3. AI Modeling for Pneumothorax

**Figure 2:**
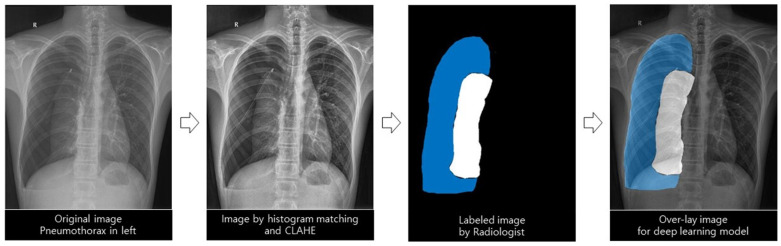
Deep learning model for automatic differentiation and segmentation of pneumothorax.

Pneumothorax labeling (true label; area ratio by simple chest radiography, gold standard; volume ratio by chest CT) was performed by a radiologist with more than 15 years of experience to develop an AI model that predicts the amount of pneumothorax and recommends a treatment method. An AI model was developed using a deep learning method trained with true labels. Subsequently, the AI-predicted pneumothorax was defined as a predicted label, and the values were compared and analyzed (Figure 2).

#### 2.3.1. Deep Learning Architecture with the Images Preprocessing Method 

This study used the U-Net architecture because of its significantly accurate medical image segmentation at various anatomical sites, including the chest [19,20]. It was used to examine and classify pneumothorax, normal lung, and other lesions presented on a simple chest radiograph. The following preprocessing steps were performed for effective segmentation. The feature values of images such as points, edges, corners, textures, and colors were more consistent through histogram matching, and contrast was enhanced through histogram equalization. Then, the brightness interval between two consecutive pixel values was adjusted using the window setting. Images with pixel normalization and resizing were used as inputs for deep learning models. We tested several hyperparameters to train the optimal deep learning model, and those with the best performance were selected. We used the Adam optimizer with a learning rate of 0.0001 and 0.85 decay, categorical cross-entropy loss, and resolution of 512 × 512 pixels (width × height).

Additionally, we used data augmentation, such as rotation, shift, and contrast change, to solve insufficient data or overfitting problems. All deep learning modeling and training procedures were implemented in DEEPPHI (http://www.deepphi.ai/, accessed on 26 July 2022), a web-based open AI platform. DEEPPHI has been used in other deep-learning-based analyses in medicine [21]. The segmented regions predicted by the AI model were used for quantification calculations.

#### 2.3.2. Quantification of Pneumothorax

**Figure 3:**
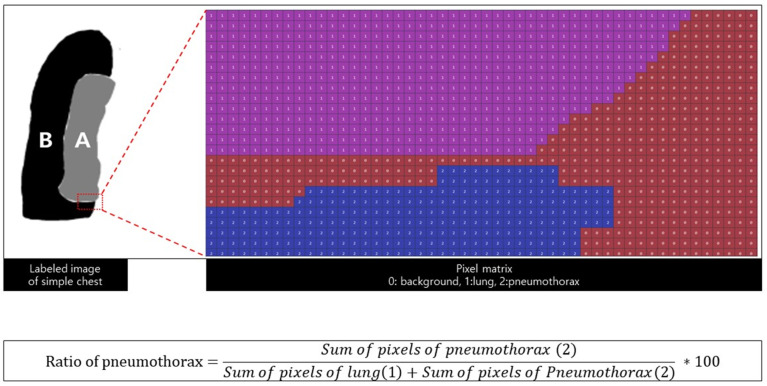
Labeling and pixel matrix A is the area of the lung, and B is the area of the pneumothorax. The area of the pneumothorax is equal to the sum of the pixel matrix, and the calculated ratio of the pneumothorax is defined as the predicted label.

Two experienced radiologists labeled the pneumothorax, normal lung, and background of the pneumothorax. The number of pixels in the labeled matrix was calculated to determine the pneumothorax amount (that is, the calculation formula) (Figure 3). The pneumothorax was measured using two types of imaging data: simple chest radiography and chest CT. First, the pneumothorax was measured on all axial images of chest CT, and they were all integrated and defined as the gold standard (volume ratio of pneumothorax). Second, the pneumothorax was measured using the same method in simple chest radiography and the result was defined as the true label (area ratio).

#### 2.3.3. Statistical Analysis

Each true and predicted label was assessed to determine whether there was a statistically significant difference from the CT label. Subsequently, the clinical decisions made by experienced thoracic surgeons were analyzed and compared according to each pneumothorax value. The normality of the measured values was determined using the Shapiro–Wilk test. Each value was compared using the paired T-test in the case of a normal distribution and the Wilcoxon signed-rank test in the case of a non-normal distribution. All statistical procedures were performed by a statistician, and Python 3.8.5 was used for calculating the pneumothorax quantification ratio and statistical analysis.

#### 2.3.4. Performance Evaluation

Accuracy, sensitivity, specificity, positive predictive value (PPV), and negative predictive value (NPV) were used to evaluate the predictive performance of the deep learning model. Additionally, the dice similarity coefficient (DSC), the most appropriate metric for evaluating the segmentation results, was used [22]. Mean absolute error (MAE) was used to compare the difference between the quantified value of pneumothorax from the CT label and each X-ray label (true and predicted X-ray labels).

## 3. Results

### 3.1. Patient Characteristics

The study included 96 patients: 77 (80%) were men, with an average age of 32 years (standard deviation of 14.57). After confirming the presence of pneumothorax using a simple chest radiograph, CT was performed. Before the CT, thoracostomy (chest intubation) was performed on 67 patients. Of the remaining 29 patients, seven underwent thoracostomy after CT, and 22 did not. The 67 patients who underwent thoracostomy before CT were defined as the training set for AI learning and the 29 patients were defined as the test set (Table 1). There should be no intervention (including chest intubation) to evaluate AI modeling that could significantly change the amount of pneumothorax between chest radiography and CT. Therefore, 29 patients who did not undergo chest intubation until the CT scan constituted the test set.

### 3.2. Classification by Deep Learning Models

According to the deep-learning-based segmentation model, the pixel-to-pixel accuracy was 97.23% for the background, 96.15% for the lung, and 97.81% for pneumothorax, with sensitivities of 99.23%, 83.57%, and 69.18%, respectively (Table 2). Additional pixels were correctly classified for the label of the lung area compared with pneumothorax. This result can be confirmed using the dice coefficient score, the harmonic average of precision, and recall. 

### 3.3. Quantification of Pneumothorax

The average amount of pneumothorax calculated by the predicted label, true label, and gold standard was 16.38% (standard deviation 6.45), 12.68% (standard deviation 8.7), and 14.85% (standard deviation 15.25), respectively. MAE was 5.41% (in true) and 8.45% (in predicted), indicating a difference of 3.03%. An experienced radiologist is more accurate in measuring pneumothorax than AI (Table 3). However, there was no statistical difference between the gold standard and the predicted label (*p* = 0.11) (Table 4). 

### 3.4. Clinical Outcomes 

Twenty-two of the 29 patients included in the test set had undergone treatments other than thoracostomy. The amount of pneumothorax in each group was approximately 10% in the gold standard and true labels; however, it was 18% in the predicted label. Thoracostomy was performed in seven, and close observation or oxygen supply was performed in 22 patients. The extent of pneumothorax was compared between the thoracostomy and other treatment groups. They were 30% in the gold standard, 18.5% in the true label, and 21.6% in the predicted label (Table 5). The number of cases in which the amount of pneumothorax exceeded 20% was seven in the gold standard (thoracostomy, *n* = 3; 43%), five in the predicted label (thoracostomy, *n* = 3; 60%), and five in the true label (thoracostomy, *n* = 3; 60%).

## 4. Discussion

It is generally accepted that the amount of pneumothorax is ‘small’ when accumulated air in the pleural cavity accounts for less than 20% of the total volume of hemithorax, which can then be observed without intervention [23]. Previous studies have proposed various methods for estimating the amount of pneumothorax, but these methods provide estimation rather than exact quantification. Kircher and Swartzel [24] drew a rectangle from a reference point to demarcate the contours of the hemithorax and the lung and subtracted one from another to find the percent pneumothorax. Some studies have also exploited deep learning techniques, but the experiments were limited to a single imaging modality; Islam et al. [25] used X-ray images, while Rohrich et al. [26] used CT images. To the best of our knowledge, this is the first study to focus on quantifying the proportion of pneumothorax through a deep learning model, cross-checking measurements from chest radiographs and CT, and providing concise parameters that can facilitate treatment decision-making. 

The predictions by the AI model developed using labeling data measured by an experienced radiologist, in terms of the presence and amount of pneumothorax, were in agreement with the real-world practice of thoracic surgeons. The pneumothorax amount calculated by the AI model was not statistically different from that estimated using CT imaging, defined as the gold standard. Thus, the AI model can make accurate predictions without CT imaging. This helps determine the appropriate clinical treatment, such as immediate chest intubation. The MAE, the amount of pneumothorax labeled by the radiologist, was more accurate than that labeled by the AI. However, AI is always available, and there is no fatigue even with repeated measurements [14]. Therefore, it may play a role in an emergency requiring immediate pneumothorax diagnosis and decision-making when the radiologist is absent on duty [27].

Notably, this model agrees with the real-world practice of experienced thoracic surgeons. Chest intubation was determined by considering the pneumothorax amount and clinical symptoms. A chest may be intubated if the amount of pneumothorax is more than 15% [28], the distance from the apex to the cupola is more than 3 cm [16], and a visible rim of over 2 cm exists on the lung margin and chest wall at the level of the hilum [15]. However, the decision is based on each surgeon’s experience due to difficulties in the calculation, inaccuracy, and usage [29]. The average amount of pneumothorax for thoracostomy was 30% in the gold standard, 19% in the true label (radiologist), and 22% in the predicted label (AI). Therefore, in actual clinical practice, chest intubation is performed when the pneumothorax is approximately 30% based on CT; however, according to AI, more than 22% can be considered an indication for chest intubation. Therefore, this model agrees with the clinical guidelines provided by the thoracic surgeons in the hospital; 20% of pneumothorax by the predicted label is used as the basis for treatment. This model imitates the decision-making of an experienced thoracic surgeon. Notably, when treatment other than thoracostomy was performed, the human and gold standard showed similar pneumothorax rates (10%). However, AI overestimated (14%); therefore, caution is required in interpretation.

This study has several limitations. First, the training set was small (67 patients). However, because the radiologist labeled all axial CT images for all patients, the actual labeling case included more than 6000 images. The model is expected to be more suitable for screening if sensitivity is improved by increasing the number of training cases in the future. Second, owing to the limited data availability, the test set was limited in size, primarily patients with a small amount of pneumothorax. Additional validation is required to determine whether it is also effective for patients with large pneumothorax. Chest intubation is a treatment of choice for patients with large pneumothorax or prominent symptoms. Therefore, although this model was validated for a small amount of pneumothorax, it has clinical value. Finally, because this was a retrospective study, selection bias could not be excluded. A more accurate model can be created using a large-scale prospective study. Further research is required to improve the sensitivity and practicality of this method.

AI can be useful in actual clinical practice. As it has a fixed value for a model that has already been trained and verified, it can be used in clinical areas and hospitals if there are only chest X-ray images as inputs. Unlike physicians, who are human and could make mistakes if they are tired or stressed, an AI would not. A well-made AI could help physicians detect and manage pneumothorax and would become most useful in extreme situations. It could, therefore, improve the clinical results and increase the HRQoL.

This paper proposed a deep-learning-based method that supports treatment decisions based on the segmentation and quantification of pneumothorax on images. The predictive value based on chest radiographs reflects the actual amount of pneumothorax and correlates well with real-world practice by expert thoracic surgeons. 

## Figures and Tables

**Table 1:** Demographic information of the study population.

Variables	Value
No. of patients	96
Age	32.85 ± 14.57
Sex	
Male	77 (80.2%)
Female	19 (19.8%)
**Manufacturers**	
Philips Medical Systems	50 (52.1%)
GE Healthcare	31 (32.3%)
DongKang	10 (10.4%)
FUJIFILM Corporation	3 (3.1%)
Samsung Electronics	2 (2.1%)
**Thoracostomy before chest CT**	
Yes (training set)	67 (69.8%)
No (test set)	29 (30.2%)

**Table 2:** Results of deep-learning-based automatic region-segmentation models.

Class	Accuracy	Sensitivity	Specificity	PPV	NPV	DSC
Background	97.23	99.23	89.64	97.32	96.85	98.26
Lung	96.15	83.57	98.97	94.78	96.41	88.83
Pneumothorax	97.81	69.18	98.56	55.92	99.18	61.84

All values are expressed as %, positive predictive value (PPV), the negative predictive value (NPV), and dice similarity coefficient (DSC).

**Table 3:** Amount of pneumothorax calculated by humans and AI.

Variables	Ratio of Pneumothorax	MAE Compared to CT
Gold standard (CT)	14.85 ± 15.25	-
True label (chest radiograph)	12.68 ± 8.7	5.41
Predicted label (calculated by AI)	16.38 ± 6.45	8.45

MAE, mean absolute error.

**Table 4:** Comparison between gold standard, predicted, and true labels.

Variables	Shapiro–Wilk Test		Wilcoxon Signed-Rank Test	
	Statistics	*p*-Value	Statistics (W) (*p*-Value *)	
Gold standard	0.7221	<0.05	144 (0.11)	-
Predicted label	0.7935	<0.05		98 (<0.05)
True label	0.8299	<0.05	-	

* *p*-value for comparison between the gold standard and predicted labels.

**Table 5:** Amount of pneumothorax between thoracostomy and other treatments.

Variables	Thoracostomy	Other Treatments
Gold standard	30.0 ±22.5/Median: 28.8	10.0 ± 7.9/Median: 8.0
True label	18.5 ± 11.9/Median: 14.8	10.8 ± 6.6/Median 8.4
Predicted label	21.6 ± 9.7/Median 19.2	14.7 ± 4.1/Median 13.6

## Data Availability

Not applicable.
